# A variant in *RESF1* is associated with Addison’s disease and multiple autoimmune syndrome in young Nova Scotia Duck Tolling Retrievers

**DOI:** 10.1038/s41598-026-42994-y

**Published:** 2026-03-12

**Authors:** Emily Brown, Scarlett Varney, Amy Young, Zena Wolf, Oded Foreman, Claire M. Wade, Angela Hughes, Anita Oberbauer, Noa Safra, Kerstin Lindblad-Toh, Shelley Burton, Danika Bannasch

**Affiliations:** 1 Department of Population Health and Reproduction, School of Veterinary Medicine, University of California, Davis, CA USA; 2 Graduate Group of Integrative Pathobiology, School of Veterinary Medicine, University of California, Davis, CA USA; 3 Center for Equine Health, School of Veterinary Medicine, University of California, Davis, CA USA; 4 LabCorp, Westborough, MA USA; 5 Genentech, South San Francisco, CA USA; 6 School of Life and Environmental Sciences, University of Sydney, Sydney, NSW Australia; 7 Department of Animal Science, University of California, Davis, CA USA; 8 Present Address: Mars Petcare, Franklin, TN USA; 9 Zoetis, Parsippany, NJ USA; 10 Department of Medical Biochemistry and Microbiology, Uppsala University, Uppsala, Sweden; 11 SciLifeLab, Uppsala University, Uppsala, Sweden; 12 Broad Institute of MIT and Harvard, Cambridge, MA USA; 13 Department of Pathology and Microbiology, Atlantic Veterinary College, University of Prince Edward Island, Charlottetown, Canada

**Keywords:** Hypoadrenocorticism, Juvenile, Canine, Inherited disease, GWAS, Diseases, Genetics, Immunology

## Abstract

Addison’s disease (AD) results in glucocorticoid and mineralocorticoid deficiencies and is often immune-mediated. While AD is uncommon in dogs, Nova Scotia Duck Tolling Retrievers (NSDTRs) exhibit increased incidence, suggesting genetic predisposition. Detailed clinical evaluation of 24 juvenile-onset cases revealed that while all dogs presented with adrenal insufficiency, at least 10 dogs (41.7%) had concurrent autoimmune conditions. This suggests juvenile-onset AD in NSDTRs represents part of a broader multiple autoimmune syndrome (MAS) with variable expressivity. Strikingly, NSDTRs affected by juvenile-onset AD had severely decreased lifespans, with a median survival of 2 years despite appropriate treatment. Genome-wide association identified a significant association on chromosome 27 (chr27:29,724,286, *p* = 6.96 × 10^− 13^). Whole-genome, short-read sequencing identified a recessive missense variant in *RESF1* (Chr27:29,736,795). The variant exhibited 76% penetrance for early-onset disease, and the decreased penetrance was not attributable to differences in Dog Leukocyte Antigen (DLA) haplotypes. Immunohistochemistry confirmed T cell infiltration in the adrenal cortex of two unrelated affected dogs, with necropsy findings including severe bilateral lymphocytic adrenalitis, multisystemic granulomatous inflammation, and lymphoplasmacytic conjunctivitis supporting autoimmune pathogenesis. This study identifies *RESF1* as a novel gene associated with autoimmune disease in NSDTRs, ranging from isolated juvenile-onset AD to multi-organ autoimmune manifestations. The findings represent a rare example of monogenic autoimmune disease and establish *RESF1* as a candidate gene for further investigation of immune tolerance mechanisms.

**Supplementary Information:**

The online version contains supplementary material available at 10.1038/s41598-026-42994-y.

## Introduction

Autoimmune diseases commonly affect individual organ systems, with conditions such as hypoadrenocorticism (Addison’s disease, or AD; OMIA: 000519-9615, OMIM %240200^1,2^) representing a well-characterized single-organ disorder in humans and dogs. AD results from deficient production of glucocorticoids and mineralocorticoids by the adrenal gland and, in humans, is often caused by autoimmune adrenalitis, which leads to destruction of the adrenal cortex^3^. In dogs, an immune-mediated etiology is also suspected, supported by immune infiltration of the adrenal cortex and atrophy of the adrenal gland at necropsy^3–6^.

While many autoimmune diseases affect single organs, around 25% of patients with autoimmune diseases will develop additional autoimmune conditions^7^. Autoimmune AD may occur in isolation, although around 50% of human AD cases are associated with additional organ-specific autoimmune disorders, including type 1 diabetes, autoimmune thyroid disease, or celiac disease^3,8^. Multiple autoimmune syndrome (MAS) is defined as the coexistence of three or more autoimmune diseases in a single patient^7^. Most MAS cases are believed to result from complex interactions between genetic and epigenetic factors and environmental triggers, although rare monogenic syndromes, such as Autoimmune Polyendocrine Syndrome type 1 (APS-1), caused by variants in the *AIRE* gene, and Immunodysregulation Polyendocrinopathy Enteropathy, X linked (IPEX), caused by variants in *FOXP3*, demonstrate that single-gene defects can also lead to multi-organ autoimmune disease. Even within these monogenic syndromes, the clinical presentation varies considerably between patients. For example, individuals with identical *AIRE* variants may present with different numbers and types of autoimmune manifestations, and the clinical variability can be so extreme that siblings with the same variants may have different phenotypes due to incompletely understood modifying factors^9,10^. Because of this variability, for APS-1, the presence of a single classic autoimmune manifestation is sufficient for diagnosis when a sibling has confirmed disease, highlighting how genetic penetrance and expressivity can vary even within families^11^. Like humans, dogs can spontaneously develop multiple autoimmune disorders. However, MAS has not been clearly defined in veterinary medicine, and the criteria for three or more autoimmune diseases may be too restrictive given the practical constraints of veterinary clinical practice, including variable diagnostic workups and owners’ financial constraints. The occurrence of multi-organ autoimmune disease in specific dog breeds provides an opportunity to investigate the genetic basis of these syndromes.

While AD can occur in a dog of any breed at any age, certain breeds, including Nova Scotia Duck Tolling Retrievers (NSDTRs), have an increased incidence of the disease^12^, suggesting a genetic component to AD. The estimated prevalence of canine AD across breeds ranges from 0.3 to 1.1%^13,14^, although certain breeds, such as Standard Poodles, Portuguese Water Dogs, and Bearded Collies, have a prevalence as high as 10%^12,15–17^. The average age of onset of AD across breeds is 4 years; however, in a study of 25 NSDTRs with AD, the average age was 2.6 years^12,18^. A previous report identified particularly young, closely related NSDTRs with juvenile-onset AD and other severe autoimmune comorbidities, including necrotizing polyarteritis and lymphocytic meningitis^19^. The NSDTR breed exhibits low within-breed genetic diversity, with an inbreeding coefficient of 0.26, supporting its use in genome-wide association studies^20^. NSDTR samples for our study were initially collected based on the diagnosis of juvenile-onset AD. However, the severe presentations and autoimmune comorbidities previously reported in young NSDTRs raised the possibility that juvenile-onset AD in this breed may represent a manifestation of MAS rather than isolated adrenal disease. In this study, we focus on NSDTRs diagnosed with AD at less than 1 year of age and utilize genome-wide association analysis, followed by whole-genome sequencing, to investigate the genetic basis of juvenile-onset AD and the potential for a naturally occurring canine model of MAS.

## Results

### Data collection

**Fig. 1:**
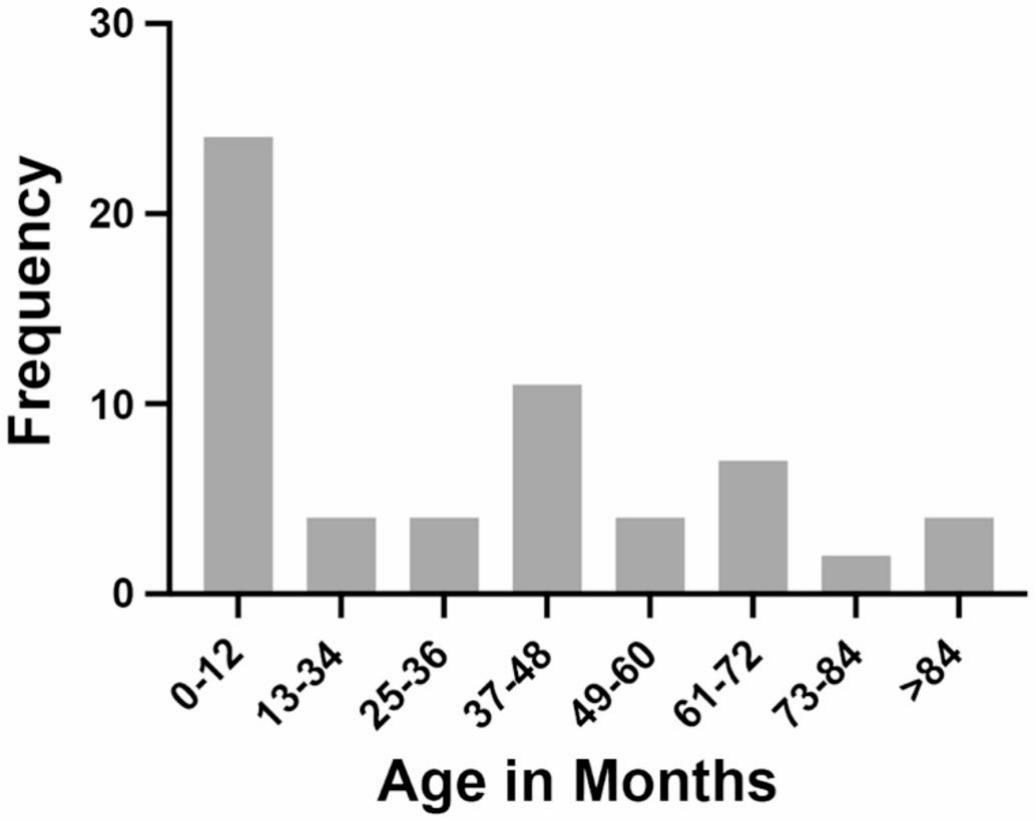
Distribution by age (in months) of AD diagnosis in collected NSDTR cases (N = 60).

Over twelve years (2004–2016), NSDTR breeders worldwide interested in supporting research into the genetic cause of AD submitted DNA samples. Sixty DNA samples from affected NSDTRs, along with the age at diagnosis, were collected. Of these, 38 included ACTH stimulation results, a confirmatory test for AD. Age at diagnosis ranged from 15 weeks to 10.5 years, with 40% of dogs (24/60) diagnosed under 1 year of age (Fig. 1). Given the unusually high frequency of juvenile cases, phenotypic and genetic evaluation focused on the subset of young dogs. DNA samples from these 24 juvenile-onset cases were collected from five countries: the United States, Canada, Denmark, Sweden, and the United Kingdom. Among these cases, three pairs of full-siblings and one half-sibling relationship were identified. Samples from parents and unaffected siblings were also collected where possible to facilitate genetic analysis. Detailed sample information, including country of diagnosis and immediate familial relationships of the 24 juvenile-onset cases, is provided in Supplemental Data 1. Pedigrees for the cases are available in Supplemental Data 2. Of the young cases, 17 had accompanying ACTH stimulation results. Fourteen of the dogs had sufficient DNA available for GWAS.

AD was reported as the sole clinical condition in only 5 of the 24 dogs diagnosed at 12 months or younger. The remaining 19 dogs exhibited additional diseases (Table 1). While 10 of the 24 juvenile-onset AD dogs (41.7%) were diagnosed with definitive autoimmune comorbidities (immune-mediated hemolytic anemia, immune-mediated thrombocytopenia, inflammatory bowel disease, stomatitis, immune-mediated polyarthritis, hypothyroid, and exocrine pancreatic insufficiency), this likely underrepresents the true prevalence of autoimmune disease in this cohort. Several dogs presented with conditions that may have autoimmune etiologies but were not extensively investigated, including conjunctivitis (n = 6), azotemia, renal failure, or pyelonephritis (n = 4), epilepsy (n = 2), keratoconjunctivitis sicca (n = 1), and arthritis (n = 1). If these diseases are included, then 70.8% of the dogs had additional morbidities. Additionally, the owner of dog 15 elected euthanasia at the time of AD diagnosis, which was at 7 weeks of age, due to the anticipated poor prognosis associated with juvenile-onset AD in this breed, precluding assessment of additional autoimmune manifestations. This broader autoimmune phenotypic spectrum contrasts with that observed in adult-onset cases (> 12 months at diagnosis, n = 36), in which only five dogs were reported to have comorbidities, all of which were limited to hypothyroidism, the most common autoimmune disease in dogs.

**Table 1:** Reported health issues for each juvenile-onset AD case in which medical records were available. Abbreviations: KCS: Keratoconjunctivitis sicca; IMHA: immune-mediated hemolytic anemia; IMTP: immune-mediated thrombocytopenia; IMPA: immune-mediated polyarthritis; IBD: inflammatory bowel disease.

Dog	Ophthalmologic	Gastrointestinal	Immune-Mediated	Neurologic	Other
1	KCS		IMHA, IMTP		
2	Conjunctivitis		IMTP		
3	Conjunctivitis, uveitis	Megaesophagus	IBD		Vaginitis
4	Conjunctivitis				
5	Conjunctivitis, uveitis		IBD, stomatitis		Umbilical hernia
6		Pancreatic abscess			Azotemia
7		Hepatitis	IMPA		
8	Conjunctivitis			Epilepsy	
9				Epilepsy	
10			Hypothyroid		Pyelonephritis
11			IMHA		
12					Pulmonic stenosis, acute renal failure, lymphoma
13					Umbilical hernia, arthritis
14					Azotemia
15					
16			Exocrine pancreatic insufficiency		
17					Heart murmur
18					
19					
20					
21	Conjunctivitis		IBD		
22			Stomatitis		
23		Small intestinal bacterial overgrowth			
24					

For 14 of the 24 dogs diagnosed with AD before one year of age, the age of death was recorded. Despite receiving appropriate treatment for AD, these 14 dogs had an average lifespan of 3.4 years with a median age of 2.0 years. The cause of death in these cases was often attributed to the other immune-mediated conditions rather than Addisonian crises or inadequate management of the disease itself. The severity and complexity of the multi-organ disease were exemplified by two full siblings, dogs 5 and 6. Dog 6 died at 4 months of age from acute complications, including pancreatitis and peritonitis, while dog 5 survived to 24 months despite requiring intensive supportive care for widespread autoimmune disease. In Burton et al., five related cases of juvenile-onset AD are described, including two littermates (cases 1 and 2) whose medical history included diffuse corneal edema, seizures, bronchointerstitial pneumonia, and necrotizing polyarteritis in addition to AD diagnosed at 17 weeks of age^19^. Necropsy information for dogs 5 and 6 from our study, as well as cases 1 and 2 from Burton et al., is reported (Table 2). These findings support the hypothesis that juvenile-onset AD in NSDTRs represents a manifestation of MAS rather than isolated adrenal disease. The variable clinical presentation, ranging from isolated AD to multi-organ autoimmune disease, likely reflects incomplete penetrance and variable expressivity of the same underlying genetic predisposition, but may also be due to differences in follow-up care and diagnostic evaluation pursued in pet dogs.

**Table 2:** Postmortem findings in juvenile-onset AD cases with available necropsy data.

Dog	Age of death (months)	Ophthalmologic	Gastrointestinal	Endocrine	Other
5	24	Perforating corneal ulcer with iris prolapse and anterior uveitis; lymphoplasmacytic conjunctivitis	Hepatitis, splenitis, and pancreatitis characterized by chronic multifocal granulomatous inflammation	Severe bilateral adrenal cortical atrophy	Pneumonia, nephritis, lymphadenitis, myocarditis, myositis, peritonitis, and encephalitis characterized by chronic multifocal granulomatous inflammation; ulcerative stomatitis, cheilitis, and glossitis; follicular and dermal atrophy with hyperkeratosis
6	4		Fibrinosuppurative pancreatitis	Severe bilateral lymphocytic adrenalitis	Peritonitis
Burton et al. Case 1^19^	3			Severe bilateral adrenal cortical atrophy	Necrotizing polyarteritis, bronchointerstitial pneumonia
Burton et al. Case 2^19^	3	Bilateral neutrophilic keratitis; lymphocytic conjunctivitis		Severe bilateral adrenal cortical atrophy	Lymphocytic meningitis

### Immunohistochemistry

**Fig. 2:**
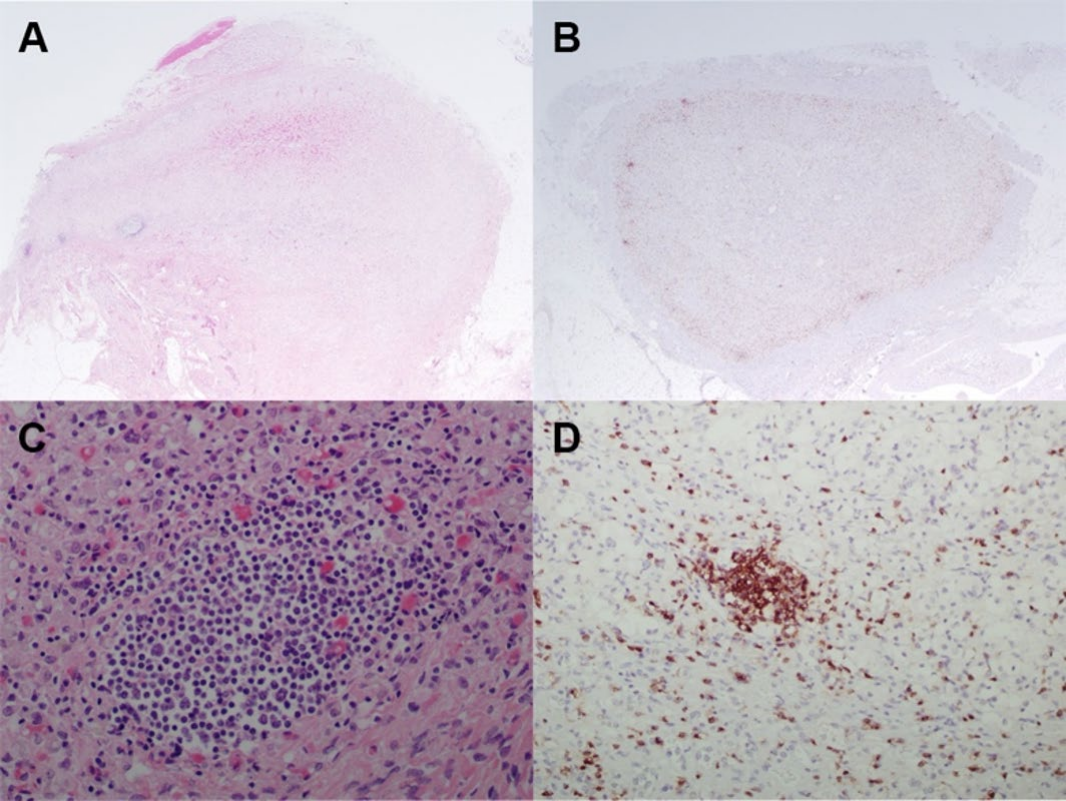
Staining of formalin-fixed adrenal tissues of a juvenile-onset AD case. **A**, **C** Aggregates of lymphoid cells in the adrenal cortex at low and high magnification, respectively. Hematoxylin and eosin (HE). **B**, **D** Immunohistochemistry of lymphocytes present in the adrenal cortex is supportive of T cells, low and high magnification, respective. CD3 immunohistochemistry.

To investigate the potential autoimmune basis of juvenile-onset Addison’s disease, immunohistochemistry was performed on adrenal gland sections from a 4-month-old affected NSDTR (case 1 from Burton et al.^19^). Hematoxylin and eosin staining shows lymphoid aggregates localized to the adrenal cortex (Fig. 2). Diaminobenzidine positive cells with anti-CD3 staining confirm these lymphoid aggregates are T cells. A T cell positive infiltrate in the adrenal gland is highly suggestive of an autoimmune disease process.

### Genome-wide association study

**Fig. 3:**
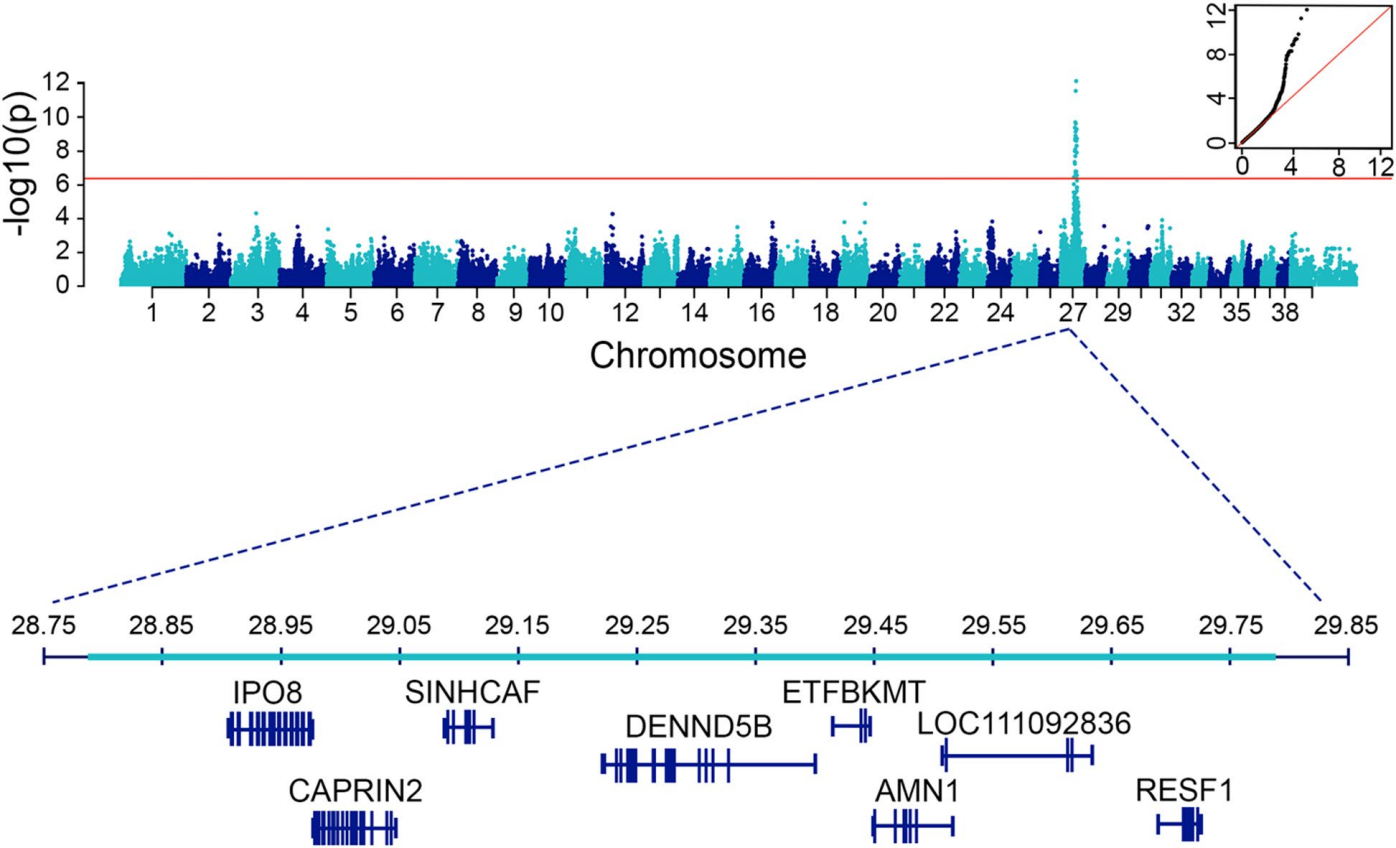
GWAS was performed using 14 cases and 33 control dogs. The Manhattan Plot of -log_10_ of p-values (y-axis) for each of the genotyped single nucleotide polymorphisms (SNPs) by chromosome (x-axis) illustrates the strong association on chromosome 27. After SNP quality control, 122,304 SNPs remained for chi square analysis. Red line denotes genome-wide significance threshold (p_Bonf._ = 4.09 × 10^–7^). The quantile–quantile (QQ) plot demonstrates observed versus expected -log(p) values. Genomic inflation ($\lambda$) is 1.11. The teal bar highlights a 1 Mb region of homozygosity on chromosome 27 (28.8 Mb-29.8 Mb; containing 8 genes) that was present in 12 of the 14 cases, with no controls homozygous for this haplotype. Of the two cases that did not share this homozygous haplotype, one was heterozygous for the haplotype and one carried two alternate haplotypes.

Genome-wide association analysis was performed using 47 NSDTRs comprised of 14 AD cases diagnosed < 12 months of age and 33 healthy controls aged > 72 months. A linear mixed model and relatedness matrix in GEMMA v.0.97 were used to account for population stratification^21^. A region of significant association was identified on chromosome 27, with the highest associated SNP at chr27:29,724,286 (canFam4, p = 6.96 × 10^–13^, genomic inflation 1.11)**.** There was an extensive region of homozygosity on chr27 extending from 28,867,342 to 29,867,310 present in 12 of the 14 cases (Fig. 3). None of the controls were homozygous for this haplotype.

### Whole genome sequencing

**Fig. 4:**
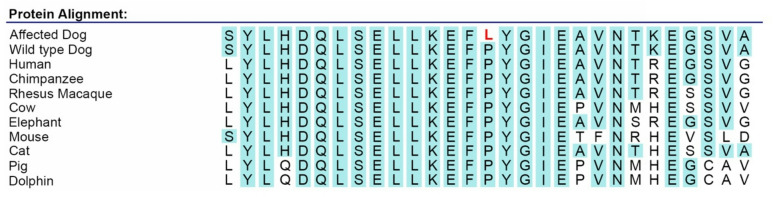
Amino acid sequence across region with RESF1 variant illustrates conservation of the altered amino acid across multiple species. The highlighted amino acids illustrate the variant’s presence in an extended region of conservation.

To investigate the associated interval further, whole-genome sequencing was performed in one NSDTR homozygous for the associated haplotype and 135 unaffected dogs without the associated haplotype. Variants (SNPs and small insertions/deletions) in the affected dog were compared to the reference set of unaffected dogs to reduce the number of potential variants in the interval to only those that were homozygous in the case. 25 variants met these criteria, including 5 intergenic variants, 18 intronic variants, and 1 variant in 3’UTR, according to SnpEff annotations^22^. Notably, only one variant was exonic and predicted to affect protein sequence, a C to T transition in *RESF1* (NC_049248.1(XM_038577211.1):c.3170C > T p.(Pro1057Leu)), resulting in an amino acid change from proline to leucine (Table 3). The canine coding sequence of this gene is 83.9% identical to human at the nucleotide level, and the canine protein is 70.7% identical to the human protein^23^. However, the altered amino acid is 100% conserved across mammals and is in a region of extended conservation (Fig. 4). The PhyloP score for this amino acid is 7.7, which is highly conserved across 470 mammalian species^24,25^. PolyPhen-2, which uses sequence conservation and protein structure to predict the effect of amino acid substitutions on protein structure and function, characterizes this variant to be damaging (score = 1.0), and CADD (Combined Annotation Dependent Depletion) predicts the amino acid change to be deleterious (score = 26.2)^26,27^. Additionally, AlphaMissense classifies the variant as likely pathogenic (score = 0.82)^28^. Because the proline to leucine missense occurs in a highly conserved region of the *RESF1* gene and this variant appears to change protein structure in a damaging way, this variant is a compelling candidate for MAS and warranted further investigation.

**Table 3:** Genic variants within region of homozygosity on CFA 27.

Location (canFam4)	Chr27:29,736,795	Chr27:28,931,007
Gene	*RESF1*	*IPO8*
SnpEff Output	Missense	3’UTR
Conservation Score (PhyloP)	7.67	-1.272

The frequency of this variant was investigated in publicly available whole genome sequence databases, and one Nova Scotia Duck Tolling Retriever was heterozygous out of 2,572 dogs^29,30^.

### Quantitative real-time PCR

*RESF1* is ubiquitously expressed at low levels in human tissues; however, there is high expression in immune tissues and cells, including CD4 + and CD8 + T cells, B cells, and NK cells^31^. To evaluate any changes in expression in *RESF1*, qRT-PCR was performed using peripheral leukocytes from 3 cases and 3 controls. All six dogs were genotyped for both variants. *RESF1* was upregulated 1.35-fold in cases compared to controls (p = 0.024). *IPO8* expression was evaluated in the same cohort. There was no statistically significant difference in expression of *IPO8* between cases and controls (p = 0.58), supporting its exclusion as a potential causative variant.

### Genotyping assay and estimation of penetrance

Of the 24 dogs affected with juvenile-onset AD, 22 were homozygous for the *RESF1* variant, 1 was heterozygous, and 1 was wild type. None of the AD cases diagnosed over 12 months of age were homozygous for the *RESF1* variant, however, 6 were heterozygous. The case used for immunohistochemistry was also verified to be homozygous for the *RESF1* variant. 328 NSDTRs not affected by AD under 1 year of age were genotyped for the missense variant in *RESF1*. Sampling of related and unrelated dogs to the original cohort used for mapping identified 7 dogs homozygous for the variant but unaffected by the disease. Each of these dogs was over 1 year of age. These dogs were therefore classified as non-penetrant cases. Based on the pedigree data and identity by descent of the variant, it is estimated that the *RESF1* variant is 76% penetrant. Genotyping also identified 109 dogs heterozygous for the variant, while the remaining 212 dogs were homozygous for the wildtype allele. Because many of the genotyped dogs were closely related to cases, the overall sample represents a biased population for allele frequency estimation. To account for this, carrier frequency was calculated using only dogs at least two generations removed from known cases, yielding a carrier frequency of 23% in this subset. Segregation of the mutation with the phenotype can be seen in the pedigrees of the cases (Supplemental Fig. 1). The probability that this segregation would occur by chance is calculated to be 3.6 X 10^–11^.

### DLA Class I and II Exon 2 typing

DLA class I genotypes and class II haplotypes were investigated as a potential mechanism mediating disease status in dogs homozygous for the *RESF1* variant. Due to limited DNA, not all clinically affected AD and non-penetrant animals were included in DLA class I and II analyses.

Eleven *DLA-88* class I allele combinations were identified in 13 NSDTRs genotyped: 7 AD cases and 6 non-penetrant animals (Table S1). A chi-square analysis was performed using 2 × 2 contingency tables to determine the significance between genotype and disease status in this cohort of dogs. It was hypothesized that *DLA-88* class I allele combinations would determine disease status in dogs homozygous for the *RESF1* variant; however, no significant association was identified between genotype and disease status (all p-values > 0.05). Using this sample set, *DLA-88* genotypes do not determine penetrance of the *RESF1* variant.

Seven DLA class II haplotypes (DLA-DRβ*x, DQβ*y, DQα*z) were identified in 16 NDSTRs genotyped: 9 AD cases and 7 non-penetrant animals (Table S2). A chi square analysis was performed using 2 × 2 contingency tables to assess significance between haplotype and disease status in this cohort of NSDTRs homozygous for the *RESF1* variant, but with differing phenotypes; however, similarly to DLA class I, no significant association between haplotype and disease status was identified (all *p*-values > 0.05). Using this sample set, a DLA class II haplotype (DLA-DRβ*x, DQβ*y, DQα*z) does not explain the incomplete penetrance of the *RESF1* variant.

## Materials and methods

### Ethics and inclusion

Collection of canine samples was approved by the University of California, Davis Animal Care and Use Committee (IACUC protocol numbers 15356, 16892, and 18561). Sample collection procedures adhered to the University of California, Davis, guidelines. This study was conducted in compliance with ARRIVE guidelines for reporting research involving animals. Owners provided written informed consent for participation in this study, including consent for blood sample collection and use of owner-provided medical records and/or pathology reports for research purposes. One dog (dog 15) was euthanized prior to being included in this study. According to the medical records, euthanasia was performed via intravenous administration of a pentobarbital-based euthanasia solution, which is an American Veterinary Medical Association-approved method for humane euthanasia in dogs. The decision to euthanize this animal was made between the owner and the personal veterinarian for the welfare of the animal. A post-mortem tissue sample was submitted by the veterinarian to our laboratory at the owner’s request for inclusion in this study. Another dog (case #1) was sampled post-mortem through an FFPE sample that was collected during necropsy as part of another study^19^.

### Phenotyping

Privately owned NSDTRs were diagnosed with AD by owner-chosen veterinarians. Owners provided ACTH stimulation results and information about age of diagnosis and other illnesses at the time of submission. To characterize the broader autoimmune phenotype, medical records were obtained for the 24 cases included in the genome-wide association study (Table 1). For two cases (dogs 5 and 6), necropsy reports were available, and information was summarized alongside necropsy information from two additional cases from Burton et al.^19^ (Table 2). There were 328 unaffected NSDTRs included in this study, based on owner-provided information at the time of blood sample submission.

### Immunohistochemistry

Adrenal tissue was collected from a deceased 4-month-old NSDTR diagnosed with AD shortly before death (case #1 from Burton et al.^19^). Formalin-fixed adrenal tissue was submitted to the UC Davis Veterinary Teaching Hospital Histology Lab for H&E and anti-CD3 staining. One of the authors (OF), a board-certified veterinary pathologist, interpreted the slides.

### Canine sample DNA extraction

DNA was predominantly extracted from buccal swabs and EDTA whole blood samples using Gentra Puregene DNA purification extraction kit (Qiagen, Valencia, CA). DNA for case #1 was extracted from FFPE tissue using Quick-DNA FFPE Miniprep kit (Zymo Research). Spectrophotometric analysis (NanoDrop ND1000, Thermo Scientific) was performed to quantify DNA concentration and assess purity prior to storage at ≤ -20˚C. Detailed sample information is provided in Supplemental Data  1.

### Genome-wide association study

Genome-wide single nucleotide polymorphism (SNP) genotyping was performed using the Illumina Canine HD 174,000 SNP array (Illumina, San Diego, CA) for 14 AD cases diagnosed less than 1 year of age and 33 healthy controls all over 6 years of age. Genotype data were initially processed and converted into PLINK format (.bed, .bim, .fam) using PLINK v.1.9.0-b.7.7^32^. SNPs were pruned from analysis if the minor allele frequency was less than 5% and the call rate was below 90%. For consistency in this publication, genomic coordinates were lifted from canFam3 to canFam4 using the UCSC liftOver tool^33^ with the canFam3ToCanFam4 chain file, retaining 122,304 SNPs. To account for population structure, a kinship matrix was generated using Genome-wide Efficient Mixed Model Association Algorithm (GEMMA) v. 0.97^21^. This matrix was subsequently used for the association analysis. A univariate linear mixed model was fitted using GEMMA, and likelihood ratio test p-values (p_lrt) were used to assess statistical significance. A Bonferroni-corrected significance threshold was calculated using the number of SNPs tested. Manhattan and QQ plots were generated using R Studio^34^.

### Whole genome sequencing and analyses

Whole genome sequencing was performed on one affected NSDTR homozygous throughout the critical interval that was also included in the GWAS and 19 unaffected NSDTRs wild type throughout the critical interval, along with 116 other unaffected dogs from varying breeds from the Bannasch Lab DNA Repository (Supplemental Data 1). Library preparation and 150 bp paired-end sequencing were performed by the UC Davis DNA Technologies Core. Whole genome sequence data were aligned to the canine reference genome CanFam4 (UU_Cfam_GSD_1.0) as previously described^35^.

The prevalence of the identified *RESF1* variant was assessed in whole genome sequence data from 1,982 apparently healthy dogs in the Dog 10 K database representing 321 breeds^30^ and 590 dogs in the Dog Biomedical Variant Database Consortium (DBVDC) representing 126 breeds^29^.

### Genotyping assay, estimation of penetrance, and segregation analysis

An additional 328 NSDTR samples were genotyped using Sanger sequencing for the *RESF1* variant identified by whole genome sequencing using 5’- GCCACAGAACCTTCCTTTGT-3’ and 5’-CACCACAACATCCAGAATCG-3’, designed using Primer3^36^. Each reaction consisted of 14.3 μl water, 2 μl 10X buffer with MgCl_2_, 1 μl dNTP, 0.8 μl of each primer, 0.1 μl AmpliTaq Gold, and 1 μl of genomic DNA. Amplified products were sequenced on an Applied Biosystems 3500 Genetic Analyzer using the Big Dye Terminator Sequencing Kit (Life Technologies, Burlington, ON, Canada) and analyzed for the polymorphism using Chromas (Technelysium Pty Ltd, South Brisbane QLD, Australia).

Using pedigree data and genotypes from available family members of affected dogs, the percent penetrance of the variant was calculated by dividing the number of dogs homozygous for the variant and affected with MAS by the total number of dogs homozygous for the variant, then multiplying by 100.

The probability that the segregation would happen by chance in these pedigrees (Supplemental Fig. 1) was calculated when genotypes for both parents were available and only on litters with full penetrance using the following formula: *p* = 0.25 for each affected offspring and p = 0.75 for each unaffected offspring.

### DLA class I genotyping

Dog Leukocyte Antigen (DLA) class I genotyping was performed on exons 2 and/or 3 of *DLA-88.* Primers for cDNA were designed as described by Ross et al.^37^ while primers for genomic DNA were designed using Primer3^36^ (Table S3). Each reaction consisted of 13.9 μl water, 2 μl 10X buffer, 1 μl dNTP, 1 μl of each primer (20 μM), 0.1 μl Hot Star Taq, and 1 μl of cDNA or genomic DNA. Amplified products were visualized on a 2% agarose gel, then sequenced on an Applied Biosystems 3500 Genetic Analyzer using the Big Dye Terminator Sequencing Kit (Life Technologies, Burlington, ON, Canada) and analyzed for heterozygous genotype calls using Chromas (Technelysium Pty Ltd, South Brisbane QLD, Australia). Sequences were queried against nucleotide sequences in BLAST (http://blast.ncbi.nlm.nih.gov/Blast) to identify the allele. *DLA-88* alleles were determined for 7 MAS cases and 6 non-penetrant MAS cases.

### DLA class II genotyping

DLA class II genotyping was performed on exon 2 of *DRβ, DQβ*, and *DQα*. *DRβ* forward primer and *DQα* forward and reverse primers were designed as recommended by Kennedy et al. (Table S4)^38^. Each reaction consisted of 14.3 μl water, 2 μl 10X buffer with MgCl_2_, 1 μl dNTP, 0.8 μl of each primer (20 μM), 0.1 μl AmpliTaq Gold, and 1 μl of genomic DNA. Amplified products were sequenced and analyzed as described above. DRβ*x, DQβ*y, DQα*z alleles and haplotypes were called for 9 MAS cases and 7 non-penetrant MAS cases, as described using for *DLA-88*.

### Quantitative real-time PCR

Primers spanning 3 exons and 2 introns were designed for *RESF1* and *IPO8* using Primer3^36^. Primers for the housekeeping gene *RPS5* spanning 2 exons and 1 intron were used as recommended by Brinkhof et al. (Table S5)^39^. A PCR protocol was employed using the Rotor-Gene SYBR Green PCR Kit (Qiagen, Valencia, CA, USA) on the Rotor Gene Q real-time PCR system: initial denaturation at 95 °C for 5 min; annealing at 95 °C for 5 s and extension at 60 °C for 10 s for 35 cycles followed by a final melt curve. Three affected MAS cases and three wild type NSDTR controls were run in triplicate with 4 ng template cDNA per reaction. The experiment was replicated three times, and 8 or 9 total technical replicates per sample were used for analysis, in which data for *RESF1* and *IPO8* were normalized to *RPS5*. REST2009 was used to determine differences in *RESF1* transcript levels between control and affected AD cases and differences in IPO8 transcript levels between control and affected AD cases^40^.

## Discussion

This study details the occurrence of juvenile-onset AD in the Nova Scotia Duck Tolling Retriever and identifies a promising causative variant of a MAS. Through genome-wide association analysis and whole-genome short-read sequencing, a missense variant in *RESF1* was identified, which resulted in a highly conserved amino acid change from proline to leucine. This alteration leads to the disease in 76% of dogs homozygous for the variant. While sample collection initially focused on juvenile-onset Addison’s disease (AD), comprehensive phenotypic characterization revealed that AD represents one manifestation of a broader autoimmune syndrome affecting multiple organ systems. The MAS phenotype has been observed in the NSDTR breed before this study, with documented findings including neutrophilic keratitis, lymphocytic conjunctivitis, necrotizing polyarteritis, and lymphocytic meningitis in addition to bilateral adrenal infiltration^19^.

NSDTRs have an increased incidence of AD compared to other breeds^12^, with affected dogs showing a younger average age at diagnosis. The long-term collection of affected dogs enabled detailed phenotyping, revealing distinct differences between juvenile-onset and adult-onset presentations. Analysis showed that the age of AD presentation ranges from 15 weeks to 10.5 years; however, the co-occurrence of additional autoimmune diseases predominantly characterized cases diagnosed before the age of 1 year. In contrast, the only comorbidity seen in adult-onset AD cases was hypothyroidism, a common autoimmune endocrinopathy in dogs^41^. Juvenile-onset AD dogs also died at younger ages, usually following complications from one of these comorbidities. This pattern suggests that juvenile-onset AD in NSDTRs is a clinical manifestation of an underlying MAS with variable expressivity rather than an isolated endocrine disorder.

Many juvenile-onset AD affected dogs present with additional immune-mediated diseases such as immune-mediated cytopenias or inflammatory bowel disease alongside AD. Of the cohort of juvenile-onset AD cases in this study, conjunctivitis was also a common concurrent disease. Conjunctivitis can represent a heterogeneous group of clinical presentations, as it is often secondary to other ocular or periocular diseases with varying causes and severities^42,43^. While in one dog (dog 4), conjunctivitis appeared uncomplicated and resolved, for the others (dogs 2, 3, 5, 8, 21), conjunctivitis was chronic or recurrent, often returning after cessation of immunosuppressive medications, suggesting it may be secondary to a more complicated autoimmune disease process. Histopathologic examination confirmed lymphoplasmacytic conjunctivitis in one affected case (dog 5), with severe ocular complications including perforating corneal ulceration, iris prolapse, and anterior uveitis documented in the same animal. The severity and complexity of MAS was particularly evident in two full siblings (dogs 5 and 6). Dog 5 developed widespread granulomatous inflammation affecting the lungs, kidneys, liver, heart, muscle, spleen, pancreas, and brain, alongside chronic oral and ocular lesions, ultimately surviving for approximately 2 years with intensive supportive care. Dog 6 presented more acutely and, despite initial clinical improvement following standard AD treatment, developed fatal complications, including pancreatitis and peritonitis, at 17 weeks of age.

Immunohistochemistry of adrenal gland tissue from case 1 from Burton et al.^19^ confirmed the presence of an inflammatory T cell infiltrate in the adrenal cortex. This dog was confirmed to be homozygous for the *RESF1* variant. Similarly, dog 6 from the current study showed severe bilateral lymphocytic adrenalitis on necropsy and was also homozygous for the *RESF1* variant. The consistent finding of autoimmune adrenalitis in two unrelated affected dogs strongly suggests an autoimmune disease process and supports the idea that a breakdown in immune system tolerance may contribute to the development of MAS in NSDTRs.

To understand the genetic basis of the disease, a genome-wide association study was performed, which identified a single genome-wide associated locus on chromosome 27, resulting in a 1 Mb homozygous haplotype in 12 of the 14 cases. The two cases that were not homozygous for the associated haplotype (nor homozygous for the later identified variant) presented with AD between 5 and 7 months of age. One of the cases was diagnosed with stomatitis and the other had no comorbidities. Given the increased incidence of AD in the breed, molecular heterogeneity of AD in NSDTRs is likely. This suggests that some dogs may develop early-onset AD without being homozygous for the identified MAS variant.

Subsequent investigation of the associated interval using whole-genome sequence data identified 25 segregating variants, of which only one was protein-coding. This missense variant was in the *RESF1* gene, which is highly expressed in lymphoid tissues and is otherwise expressed ubiquitously at low levels in other tissues in people^31^. *RESF1* appears to be conserved across species, which suggests a functional significance^44^. The variant is significantly enriched in affected individuals (92% of cases are homozygous) compared to controls (absent from 2,571 dogs representing more than 350 breeds), all computational evidence supports pathogenicity, and there is a less than 1 X 10^–8^% probability that this pattern of segregation occurred by chance. Combined with its localization to a small associated genomic interval, this evidence supports that the missense variant in *RESF1* is likely pathogenic based on current International Society of Animal Genetics criteria developed by the Variant Pathogenicity Working Group^45^, available at https://www.isag.us/committees.asp. The missense variant leads to a small but significant increase in *RESF1* expression in leukocytes of affected dogs compared to controls. This subtle change may be attributed to the fact that expression was queried across multiple white blood cell types, including lymphocytes, NK cells, and monocytes, because samples were “pooled.” The proportion of these cell types could differ between cases and controls, and if there was a substantial difference in expression in one of the cell types, it could be masked by relatively normal expression in other subtypes. Additionally, due to constraints working with owned pets, the experiment included a small sample size of only three cases and three controls per group, which could skew results. Despite increased *RESF1* transcripts in juvenile-onset AD cases, given the homozygous missense variant, the amino acid substitution may lead to a dysfunctional RESF1 protein. Alternatively, the less functional protein may elicit increased expression as a compensatory mechanism.

The role of *RESF1* in autoimmune disease has not yet been investigated. However, we hypothesize that its role in pathogenesis involves altered self-recognition and T cell differentiation during immune system development or overstimulation of the immune system by decreased repression of retroviral elements, both of which would result in increased immune activity. *RESF1*, or Retroelement Silencing Factor 1, interacts with the histone lysine methyltransferase *SETDB1* to epigenetically repress endogenous retroviral elements in embryonic stem cells^44^. *RESF1* shows highest expression in naïve regulatory T cells (Tregs), which are critical for establishing immune tolerance^31,46^. If defects in *RESF1* compromise Treg function, it could impair the immune system’s ability to distinguish self from non-self, leading to a breakdown of immune tolerance. The likely role of *RESF1* in T cell differentiation is consistent with *RESF1* expression data showing naïve T cells have higher *RESF1* expression than mature T cells^31^. In addition to its role in immune cell development, *RESF1* also functions in retroviral element suppression, and a loss of *RESF1* due to defective protein production could cause an increase in retroviral elements, which has been linked to some autoimmune diseases in humans such as systemic lupus erythematosus^47^ and Aicardi-Goutières Syndrome^48^. It has been hypothesized that the mechanism of autoimmune disease in this case is that accumulation of retroviral elements and related proteins leads to chronic or episodic systemic inflammation and IFN production triggered by repeated immune response^47^. It is possible that a similar mechanism is responsible for the phenotype observed in dogs with MAS. Additional experimentation is needed to confirm the role *RESF1* plays in the establishment of immunologic tolerance in dogs.

After identification of the variant, additional dogs related to juvenile-onset AD cases were genotyped, which resulted in identification of non-penetrant cases. Based on genotyping of collected samples, the penetrance of the *RESF1* variant was calculated at 76%, suggesting a highly penetrant autoimmune disease variant. However, this is likely an overestimate, as a large portion of tested dogs were related to juvenile-onset AD cases. Even though penetrance of the *RESF1* variant was not associated to the DLA on chromosome 12 using GWAS, class I and class II DLA genes were investigated as a mechanism to explain the incomplete penetrance of the variant. Previous studies in dogs have shown that DLA genes may mediate autoimmune disease status in diseases such as AD, hypothyroidism, and diabetes mellitus^38,49,50^. In humans, corresponding human leukocyte antigen (HLA) haplotypes have also been shown to explain the penetrance of familial type I diabetes in families^51^. However, we did not identify a significant association between DLA class I or class II haplotypes and juvenile-onset AD in dogs homozygous for the *RESF1* variant. The small sample size of this experiment limits the ability to definitively exclude DLA involvement in disease penetrance. While no clear mechanism for penetrance has been identified, it is possible that environmental triggers or epigenetic factors may play a role in determining which dogs develop the disease. Additionally, given the multiple autoimmune syndrome phenotype seen in many juvenile-onset AD cases, it is also possible that non-penetrant dogs may have autoimmune disease manifestations that do not include juvenile-onset AD.

Autoimmune diseases are a heterogeneous group of disorders that involve both genetic susceptibility factors and environmental triggers, leading to a breakdown of self-tolerance^52^. These disorders tend to be polygenic, with each gene conferring a slight increase in susceptibility, while monogenic forms, where a defect in a single gene can cause disease, are rare^52^. Monogenic autoimmune diseases provide valuable insights into the breakdown of immune tolerance, revealing essential checkpoints in immune regulation. Single-gene variants in *AIRE* and *FOXP3* can cause multi-organ autoimmune syndromes, demonstrating that defects in key immune regulatory pathways can lead to the breakdown of self-tolerance^53^. *AIRE* variants demonstrate the importance of central tolerance in the thymus, as patients with these variants can develop Autoimmune Polyglandular Syndrome type 1 (APS-1), characterized by adrenal insufficiency, hypoparathyroidism, and mucocutaneous candidiasis. *FOXP3* variants highlight the role of regulatory T cells, as patients with variants in *FOXP3* develop Immunodysregulation, Polyendocrinopathy, and Enteropathy, X-linked (IPEX), which is characterized by a group of autoimmune conditions like dermatitis and diabetes mellitus^54^. Variable expressivity and incomplete penetrance are recognized features of monogenic autoimmune diseases. Not all individuals with *AIRE* or *FOXP3* variants develop the full spectrum of autoimmune manifestations, and some may present with isolated organ-specific autoimmune disease rather than clear multiple autoimmune syndrome^55,56^. Patients with identical variants in the *AIRE* gene can develop different combinations of autoimmune conditions, and there are over 20 known phenotypes associated with APS-1^55^. The average age of onset for APS-1 is 9 years, and 67% of people with APS-1 had adrenal failure as a component of their disease^57^. APS-1 patients have a dramatically increased mortality rate compared to the general population, with median survival of between five and 34 years of age^55^. Similarly, *FOXP3* variants in IPEX syndrome also demonstrate phenotypic variability despite identical variants, including differences in age of disease onset, single- and multi-organ involvement, and mild to severe phenotypes^53,58,59^. These examples of severe, monogenic autoimmune diseases suggest that genetic background, epigenetic factors, and environmental triggers likely all play an important role in autoimmune disease expression.

The identification of *RESF1* as a causative gene for MAS represents a monogenic cause of autoimmune disease. The finding that a missense autosomal recessive variant can lead to AD in 76% of homozygous dogs highlights the important role *RESF1* plays in immune regulation. These results support *RESF1* as a potential mediator of autoimmune disease. Since identification of the *RESF1* variant and its association with the juvenile-onset AD phenotype, a genetic test has been available to NSDTR breeders through UC Davis Veterinary Genetics Laboratory (https://vgl.ucdavis.edu/test/jadd-nsdtr). As a result, the frequency of the associated allele cannot be determined in NSDTR population. However, since implementation of genetic testing, no additional juvenile-onset AD dogs from tested parents have been reported to our knowledge. The apparent elimination of the disease from the breed following the implementation of testing for this variant further supports the conclusion that the missense variant in *RESF1* is the causative variant for MAS and juvenile-onset AD in the NSDTR breed. This work establishes *RESF1* as a candidate gene for investigation in other autoimmune disease and provides insight into mechanisms of immune tolerance breakdown.

## Supplementary Information

Below is the link to the electronic supplementary material.

Supplementary Material 1

Supplementary Material 2

## Data Availability

The whole genome sequence data used in this study are available in the SRA repository, with Bioproject, Biosample, and SRR Accession numbers listed in Supplemental Data 1.
