# Supplementary material for: A variant in RESF1 is associated with Addison’s disease and multiple autoimmune syndrome in young Nova Scotia Duck Tolling Retrievers
Source: Sci Rep. 2026 Mar 12;16:13194. doi: 10.1038/s41598-026-42994-y (PMC13103380; doi:10.1038/s41598-026-42994-y)
Supplement: Supplementary file 1 — Supplementary Material 1 [file 41598_2026_42994_MOESM1_ESM.docx]

A variant in *RESF1* is associated with Addison’s disease and multiple autoimmune syndrome in young Nova Scotia Duck Tolling Retrievers

Emily Brown*, Scarlett Varney*, Amy Young, Zena Wolf, Oded Foreman, Claire M. Wade, Angela Hughes, Anita Oberbauer, Noa Safra, Kerstin Lindblad-Toh, Shelley Burton, Danika Bannasch

*These authors contributed equally to this work.

Correspondence: [dlbannasch@ucdavis.edu](mailto:dlbannasch@ucdavis.edu)

Supplemental Data 2: Supplemental_Tables_Figures


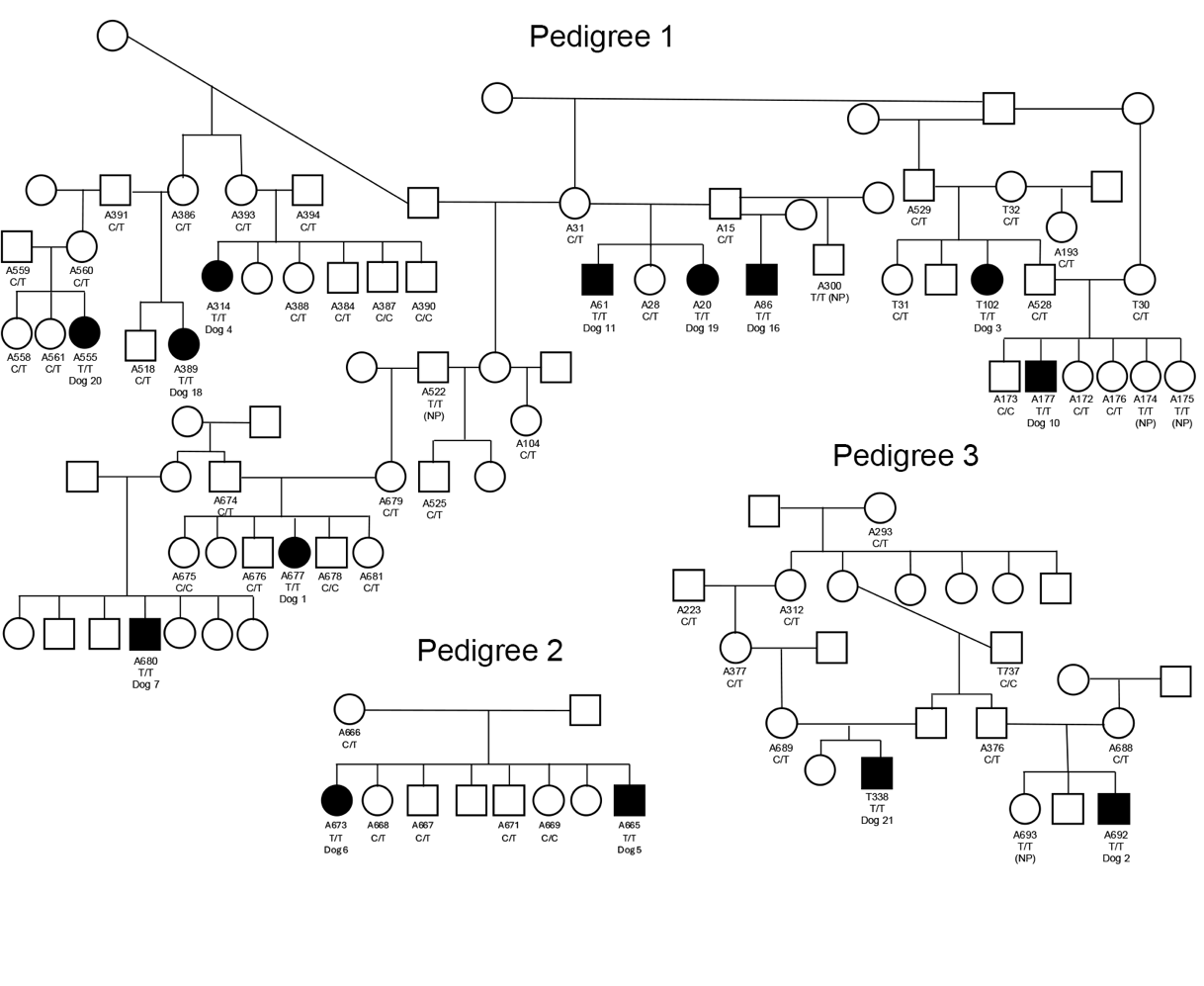

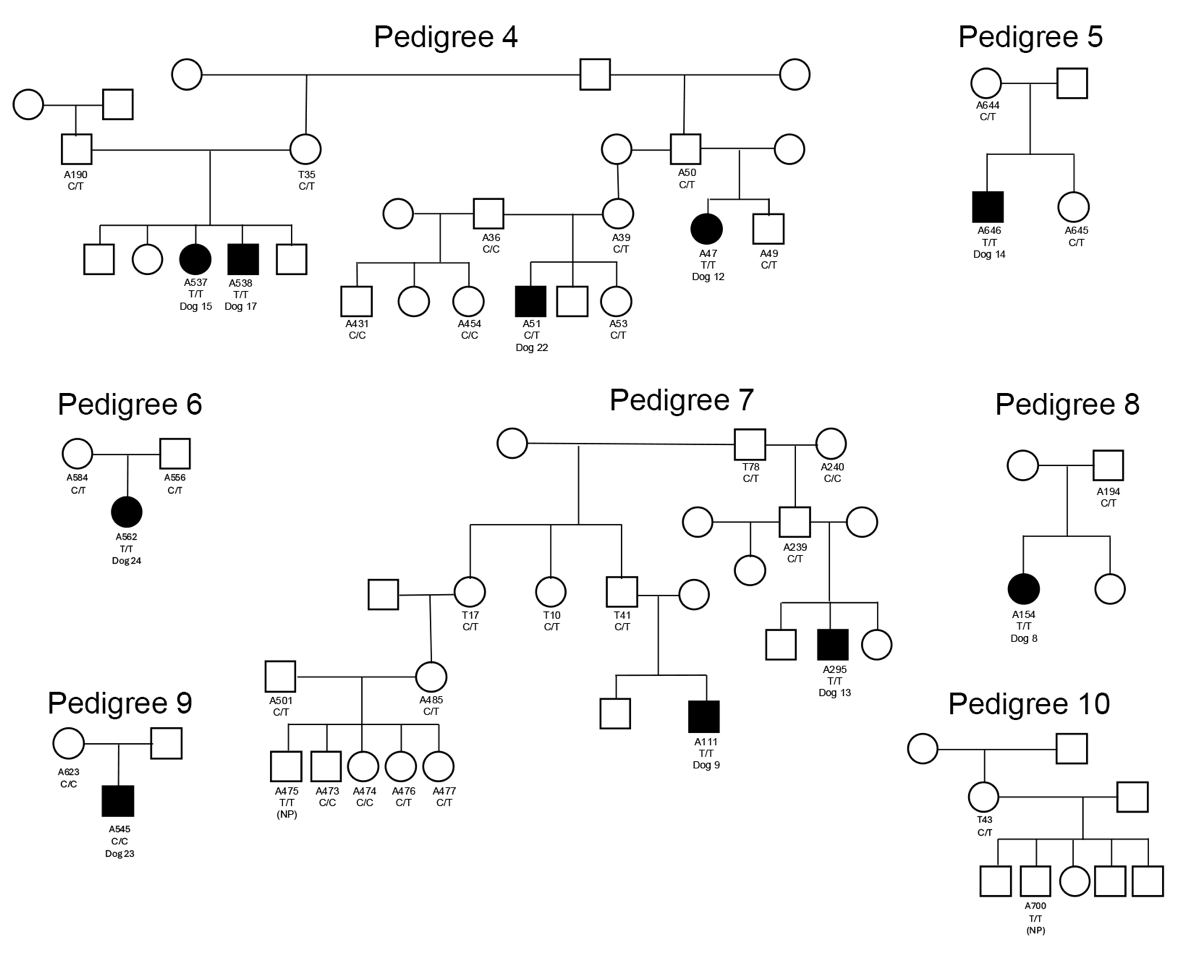


Figure S1. Pedigrees of juvenile-onset AD cases and related dogs. Genotype and, where applicable, case numbers are indicated below each symbol; dog numbers correspond to Table 1. Affected dogs are shown as filled symbols. Seven dogs were identified as non-penetrant, having a T/T genotype but no diagnosis of AD by one year of age. Two cases were not homozygous for the associated variant: A545 was homozygous wildtype and A51 was heterozygous, suggesting additional or alternative genetic contributions in these individuals.

| ***DLA-88* Genotype** | **AD (n=7)** | **NP (n=6)** | **p-value** |
| --- | --- | --- | --- |
| DLA_88*01202/ DLA88_novel | 1 | 1 | 0.34 |
| DLA_88*01202/ DLA88_X1 | 0 | 1 | 0.25 |
| DLA_88*01202/ DLA88_novel_9 | 0 | 1 | 0.25 |
| DLA_88*50801/ DLA88_novel_9 | 1 | 0 | 0.25 |
| DLA88_novel_9/ DLA88_novel | 1 | 0 | 0.25 |
| DLA88_novel_25/ DLA88_novel_25 | 1 | 0 | 0.25 |
| DLA88_novel_25/DLA88_novel_3 | 0 | 2 | 0.09 |
| DLA88_novel_25/DLA88_novel_6 | 1 | 0 | 0.25 |
| DLA88_novel_25/DLA88_novel_4 | 0 | 1 | 0.25 |
| DLA88*012:01/ DLA88*012:01 | 1 | 0 | 0.25 |
| DLA88*508:02/DLA88_novel_34 | 1 | 0 | 0.25 |

Table S1. DLA-88 genotypes present in 7 AD and 6 non-penetrant (NP) NSDTR listed with corresponding p-values. No significant associations between DLA-88 genotype and penetrance status were identified.

| **Haplotype** | ***DRβ*** | ***DQβ*** | ***DQα*** | **AD (n=9)** | **NP (n=7)** | **p-value** |
| --- | --- | --- | --- | --- | --- | --- |
| 1 | 00601 | 02001 | 005011 | 7 | 5 | 0.47 |
| 2 | 01501 | 00601 | 00301 | 2 | 1 | 0.42 |
| 3 | 01501 | 02301 | 00601 | 3 | 2 | 0.47 |
| 4 | 01502 | 02301 | 00601 | 3 | 4 | 0.29 |
| 5 | 01502 | 00301 | 00601 | 0 | 1 | 0.09 |
| 6 | 02301 | 00501 | 00301 | 1 | 1 | 0.47 |
| 7 | 00401 | 01501 | 00201 | 2 | 0 | 0.14 |

Table S2. DLA class II genotyping results for 9 AD affected NSDTRs and 7 non-penetrant (NP) NSDTRs. No significant association between DLA class II haplotype and penetrance status was identified.

|  | **Forward Primer (5’🡪3’)** | **Reverse Primer (5’🡪3’)** |
| --- | --- | --- |
| *DLA-88* cDNA primers | CGGAGATGGAGGTGGTGA | GGTGGCGGGTCACACG |
| *DLA-88* exon 3 genomic primers | TCCCGGGAGGTTTTACTTTC | GGTGTTCTGACCCTGAGTCC |

Table S3. Forward and reverse primers used to genotype *DLA-88* in 13 dogs homozygous for the *RESF1* variant: 7 dogs had MAS while 6 dogs were non-penetrant.

|  | **Forward Primer (5’🡪3’)** | **Reverse Primer (5’🡪3’)** |
| --- | --- | --- |
| *DRβ* | GATCCCCCCGTCCCCACAG | TGTGTCACACACCTCAGCACCA |
| *DQβ* | GGTTGACGGGCATCAGAG | GGTGCGCTCACCTCGCCGCT |
| *DQα* | TAAGGTTCTTTTCTCCCTCT | GGACAGATTCAGTGAAGAGA |

Table S4. Forward and reverse primers used to genotype *DRβ, DQβ,* and *DQα* in 16 dogs homozygous for the *RESF1* variant: 9 dogs had MAS and 7 were non-penetrant.

|  | **Forward Primer (5’🡪3’)** | **Reverse Primer (5’🡪3’)** |
| --- | --- | --- |
| *RESF1* | AGGATCTGCAGCCTTGTCCT | CAAATCCATCTGCACCGAAG |
| *IPO8* | GGAAGGAATAATTCGGTCTCCA | AGGCTCTCTTTCCTCTGCTTC |

Table S5: Forward and reverse primers used to measure transcript levels of *RESF1* and *IPO8* on qRT-PCR.
